# A Mummy Unrolled

**Published:** 1890-05

**Authors:** 


### A MUMMY UNROLLED.

### HOW THE ANCIENT EGYPTIANS PREPARED THEIR DEAD FOR BURIAL.

Mr. E. A. Budge, M. A., of the British Museum, recently unrolled an Egyptian mummy belonging to the University College, London. The London *Times* thus describes the process : "The mummy was closely smothered in scores of yards of thick, yellowish linen of fine texture. The bands of linen varied in width from four to five inches to about a foot. Some of them lay lengthwise along the body ; others were wrapped around it. At the beginning of the process of unrolling there was a very perceptible sickly smell of aromatics, which, as the work went on, gave place to a more pronounced and decidedly disagreeable odor. When a great part of the linen had been removed, black stains, caused by the bitumen, became apparent, and nearer the body the wrappings had suffered considerably from contact with this substance. Two small pieces of linen with fringes were discovered in the course of the unrolling, and these bore inscriptions more or less impaired by the bitumen. When at last the coverings had been removed, the body was found to be of a very dark brown color—so dark, indeed, as to be almost black. The skin where it remained was hard and shiny, the arms and hands lay lengthwise upon the abdomen, while the heart and intestines were placed beneath the knees. The features when disclosed stood out very clearly, and were those of a rather handsome person, but the sex could not be determined. Glass eyes had been placed in the head, and there was a linen plug in the ear. The mummy belonged to a period about 800 years before Christ. It was filled with bitumen, and nearly all the flesh was destroyed in consequence. Parts of the skin remained upon the breast, and the bones were in a fairly good condition. The intestines, instead of being put in pots, as they usually were in the case of persons of high birth, were placed beneath the legs. The only inscription decipherable was the name of Osiris, over the part of the stomach dedicated to that god, and a prayer for the heart of the deceased." Mr. Budge explained that there were three principal methods of preserving the human body by mummification. "The first process required that the intestines should be extracted and embalmed in four pots dedicated to four gods. The body was then soaked in natron for seventy days. At the end of that time it was washed and then carefully bandaged in hundreds of yards of linen. By the second process the intestines were simply dissolved out by means of natron, after which the body was soaked in natron and then mummified. By the third process the body was merely salted and put into a pot."

